# Narrowing of the Parent Artery Angle Is Associated With Intracranial Aneurysm Growth

**DOI:** 10.7759/cureus.51677

**Published:** 2024-01-04

**Authors:** Shinya Yamaguchi, Masato Osaki, Taisuke Kitamura, Mariya Hokazono, Kayo Wakisaka, Takako Maruyama, Chiharu Yasuda, Tesuro Sayama, Shuji Arakawa, Koji Yoshimoto

**Affiliations:** 1 Department of Neurosurgery, Steel Memorial Yawata Hospital, Kitakyushu, JPN; 2 Department of Cerebrovascular Disease, Steel Memorial Yawata Hospital, Kitakyushu, JPN; 3 Department of Neurosurgery, Kyushu University, Fukuoka, JPN

**Keywords:** risk factors, intracranial aneurysm growth, parent artery angle narrowing, long-term follow-up, magnetic resonance angiography (mra), unruptured cerebral aneurysm

## Abstract

Objective: Although risk factors for intracranial aneurysm growth have been reported, studies investigating the influence of the parent artery angle are limited. In this study, we examined the relationship between intracranial aneurysm growth and parent artery angle narrowing by analyzing long-term follow-up magnetic resonance angiography data.

Methods: We retrospectively reviewed data of patients with untreated aneurysms and those treated by simple coil embolization, who were followed up by magnetic resonance angiography for over 24 months at the Steel Memorial Yawata Hospital between August 2007 and March 2023. We investigated the relationship of aneurysm growth with parent artery angle narrowing, age, sex, follow-up duration, previous subarachnoid hemorrhage, hypertension, smoking, aneurysm location, aneurysm type, maximum size, and neck size.

Results: A total of 180 aneurysms of 162 patients (women, n=113; untreated, n=136) were included. The median age at aneurysm diagnosis was 71 (63.8-76) years and the median follow-up duration was 69 (45-120) months. Among the 180 aneurysms, 41 (untreated, n=30; treated by simple coil embolization, n=11) showed growth during the follow-up period, with a risk of 4.4%/patient-year. In the univariable analysis, the parent artery angles on the initial and last follow-up images and angle change were significantly associated with aneurysm growth. However, in the multivariable analysis, the association remained significant only for angle change (odds ratio, 2.21; 95% confidence interval, 1.42-3.45). The cutoff value of parent artery angle change for intracranial aneurysm growth was -3.4°.

Conclusion: Parent artery angle narrowing was significantly associated with intracranial aneurysm growth. This parameter may be useful for the monitoring of patients with unruptured intracranial aneurysms and may contribute to discerning the mechanism of intracranial aneurysm growth.

## Introduction

Intracranial aneurysm growth has been associated with several factors, including patient age, female sex, previous subarachnoid hemorrhage (SAH), hypertension, smoking, and aneurysm location, size, and shape [1-3]. Prior studies have also indicated a relationship between intracranial aneurysm occurrence, growth, and rupture and the configuration of the vessel containing the aneurysm [4-11]. These findings indicate that hemodynamics in the related vessels could affect aneurysm growth. However, few studies have investigated the relationship between aneurysm growth and morphological change in the parent artery. Only Boucherit et al. investigated the association between bifurcation aneurysms and the angle of the two daughter vessels (bifurcation angle) and reported that aneurysm growth is associated with changes in the bifurcation angle, specifically with widening [10].

In a previous study, we demonstrated that narrowing of the parent artery angle was associated with the recurrence of aneurysm after coil embolization [12]. In that series, we also observed the growth of recurrent aneurysms. Considering these findings, we hypothesized that the growth of untreated aneurysms and the growth and recurrence of aneurysms after coil embolization could be related to the narrowing of the parent artery angle. Therefore, in the present study, we investigated the relationship between intracranial aneurysm growth with the parent artery angle, with changes in this angle, and with patient and aneurysm characteristics by analysis of long-term follow-up data.

## Materials and methods

Study design and population

We retrospectively reviewed data of patients with untreated intracranial aneurysms and with intracranial aneurysms treated by simple coil embolization who were followed up by magnetic resonance angiography (MRA) for over 24 months at the Steel Memorial Yawata Hospital between August 2007 and March 2023. Ethics approval was obtained from the Institutional Review Board of Steel Memorial Yawata Hospital (No. 23-52). The requirement for obtaining patient consent was waived because of the retrospective study design.

Based on whether growth was observed during the follow-up period, all aneurysms were classified as growing (Group G) or non-growing (Group nG). For each aneurysm, we measured the parent artery angle and calculated its chronological change [12]. Data were compared between the groups to examine the relationship between aneurysm growth and parent artery angle and its change. In addition, we analyzed the association of the following factors with aneurysm growth: age, sex, follow-up duration, previous SAH, hypertension, smoking, aneurysm location, type (bifurcation or sidewall), aneurysm maximum size, and neck size.

MRA data processing

All MRA images were obtained using two device models, Archieva 1.5T (Koninklijke Philips Electronics NV, Amsterdam, Netherlands) and Ingenia 3T (Koninklijke Philips Electronics NV, Amsterdam, Netherlands). Time-of-flight digital imaging and communications in medicine data acquired from the two devices were transferred to the AZE Virtual Place (Cannon Medical Systems, Odawara, Tochigi, Japan) for analysis. Aneurysm measurements included height, width, depth, and neck size. Aneurysm growth was defined as a change >1 mm in each of these measurements [13]. Image comparison was performed using images obtained with the same model as much as possible.

Measurement of the parent artery angle

**Figure 1:**
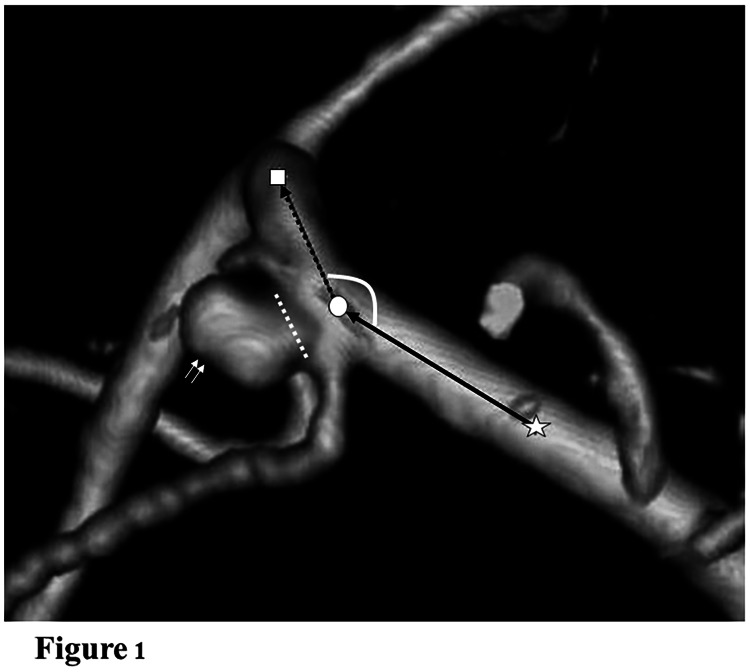
Measurement of the parent artery angle Measurement of the parent artery angle of the right middle cerebral artery aneurysm in a 40-year-old man (caudal-to-cranial view). The center of the curvature of the parent artery at the aneurysmal neck is circled in white, the center of the curvature just proximal to the aneurysm (proximal point) is noted by a white star, and the center of the curvature just distal to the aneurysm (distal point) is noted by a white square. The aneurysm is noted by two small white arrows, and the neck of the aneurysm is indicated by small white dots. The proximal main inflow (middle cerebral artery first segment flow) is indicated by a black arrow, and the distal main outflow (middle cerebral artery second segment flow) is indicated by a black dotted arrow. The parent artery angle was 145.1°.

The parent artery angle was defined based on the main inflow and outflow angles. For bifurcation aneurysms, if the outflow arteries had the same diameter, that with a wider angle was selected as the main outflow artery, and if the bifurcation aneurysm was mounted on the daughter vessel, this vessel was selected as the main outflow. The parent artery angle was measured from the direction where the proximal (inflow) artery, distal (outflow) artery, and aneurysm were observed in the same plane. The image direction was kept consistent for each measurement. The parent artery angle was measured in Adobe Photoshop (Adobe Systems, Mountain View, CA) using three points: the center of the curvature of the parent artery at the aneurysmal neck; the center of the curvature just proximal to the aneurysm, as the proximal point; and the center of the curvature just distal to the aneurysm, as the distal point, allowing for a semi-automatic measurement of the vascular angle (Figure 1).

Statistical analysis

Statistical analyses were performed using JMP® 14 (JMP Statistical Discovery LLC, Cary, NC). Between-group comparisons were performed using Fisher’s exact test for categorical variables and Wilcoxon’s rank-sum test for continuous variables. Aneurysm location was analyzed by Pearson’s chi-squared test. P-values of <0.05 were considered to indicate statistical significance. Univariable analyses were employed to identify risk factors for aneurysm growth. Variables with significant differences in the univariable analysis were further analyzed by multivariable analysis. The prognostic performance of the identified risk factors for aneurysm growth was analyzed using receiver operating curve analysis.

## Results

Patient characteristics

A total of 180 aneurysms of 162 patients (women, n=113; untreated, n=136) were included in the analysis. The median age at aneurysm diagnosis was 71 (63.8-76) years and the median follow-up duration was 69 (45-120) months. Accordingly, this study included 931.5 patient-years. Aneurysm growth was observed in 41 aneurysms of 41 patients, with a risk of 4.4%/patient-year. The breakdown of growth aneurysms is as follows. Thirty untreated aneurysms showed continued growth during follow-up. Among the eleven simple coiled aneurysms, nine aneurysms continued to grow and recurred after simple coil embolization along with narrowing of the parent artery angle, while two aneurysms that showed growth before treatment were stable after simple coil embolization. Groups G and nG included simple coiled aneurysms (Group G, 26.8%; Group nG, 23.7%), with no significant difference in the rate of treated aneurysms between groups (P=0.6833).

Risk factors for aneurysm growth

In the univariable analyses, the parent artery angle on the initial image, the parent artery angle on the last image, and the parent artery angle change were significantly associated with aneurysm growth (Table 1). Growing aneurysms had narrow parent artery angles and demonstrated a narrowing change of the parent artery angle during the follow-up period.

**Table 1:** Univariable analysis of the characteristics of the patients in our cohort *Values are presented as median (interquartile range). †Statistically significant. Abbreviations: SAH=subarachnoid hemorrhage, ICA=internal carotid artery, MCA=middle cerebral artery, ACA=anterior cerebral artery

Variable	Group G	Group nG	P value
Number of aneurysms	41	139	-
Age (years)*	72 (62.5–78.5)	70 (64–75)	0.3621
Female (%)	82.9	66.9	0.0528
Follow-up duration (months)*	86 (50–112)	65 (44–120)	0.1757
Previous SAH (%)	12.2	6.5	0.3156
Hypertension (%)	95.1	87.1	0.2553
Smoking (%)	17.1	22.3	0.5226
Aneurysm location (%)			0.2499
ICA	29.3	41.7	
MCA	22.0	26.6	
ACA	29.3	19.4	
Posterior circulation	19.5	12.2	
Bifurcation type (%)	56.1	41	0.1079
Maximum diameter of aneurysm (mm)*	4.6 (3.6–5.9)	3.9 (3.0–5.2)	0.0976
Neck diameter of aneurysm (mm)*	3.4 (2.5–4.1)	3.0 (2.5–3.7)	0.1101
Parent artery angle at initial image (°)*	108.5 (84.3–122.8)	136.2 (101.4–154.6)	0.0003^†^
Parent artery angle at last image (°)*	101.4 (74.5–116.0)	137 (101.5–156.1)	<0.0001^†^
Parent artery angle change (°)*	-7.3 (-12.7 to -5.0)	0.2 (-1.1 to 1.9)	<0.0001^†^

In the multivariable analysis, only the change in the parent artery angle remained significantly associated with intracranial aneurysm growth (Table 2), with an odds ratio of 2.21 (95% confidence interval, 1.42-3.45).

**Table 2:** Multivariable analysis of risk factors for aneurysm growth †Statistically significant. CI=confidence interval

Variable	Odds ratio	95% CI	P value
Parent artery angle at initial image (°)	1.03	0.73-1.46	0.7997
Parent artery angle at last image (°)	0.99	0.70-1.40	0.9343
Parent artery angle change (°)	2.21	1.42-3.45	0.0005^†^

**Figure 2:**
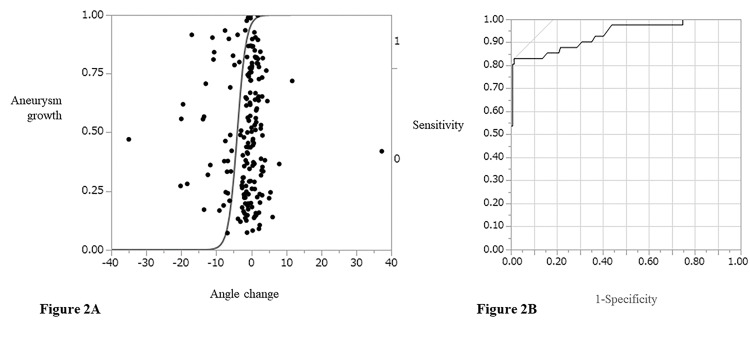
Logistic plot (A) and receiver operating characteristic curve (B) of parent artery angle change and intracranial aneurysm growth The area under the curve was 0.934.

**Figure 3:**
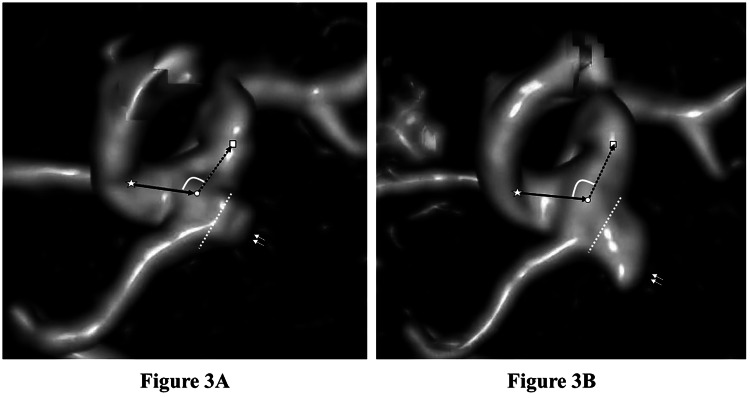
Representative images of a sidewall-type growing aneurysm of the left internal carotid artery-posterior communicating artery in an 83-year-old woman (caudal-to-cranial view) The median follow-up duration was 39 months, with evident aneurysm growth. The center of the curvature of the parent artery at the aneurysmal neck is circled in white, the center of the curvature just proximal to the aneurysm (proximal point) is noted by a white star, and the center of the curvature just distal to the aneurysm (distal point) is noted by a white square. The aneurysm is noted by two small white arrows, and the neck of the aneurysm is indicated by small white dots. The proximal main inflow (internal carotid artery flow before posterior communicating artery bifurcation) is indicated by a black arrow, and the distal main outflow (internal carotid artery flow after posterior communicating artery bifurcation) is indicated by a black dotted arrow. The parent artery angle was 116.5° on the first (A) and 109.6° on the last magnetic resonance angiography image (B).

**Figure 4:**
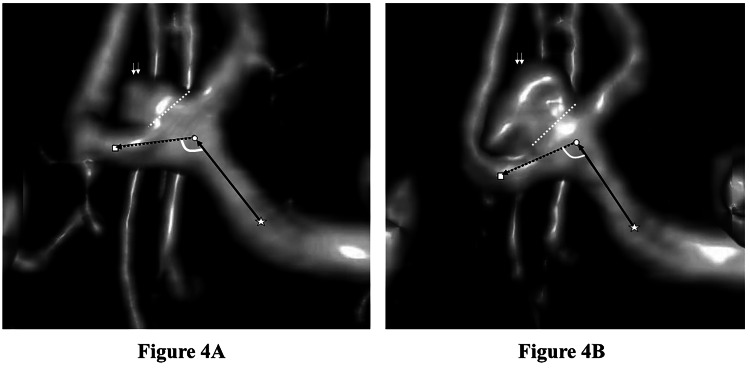
Representative images of a bifurcation-type growing aneurysm of the anterior communicating artery in a 64-year-old woman (caudal-to-cranial view) The median follow-up duration was 105 months, with evident aneurysm growth. The center of the curvature of the parent artery at the aneurysmal neck is circled in white, the center of the curvature just proximal to the aneurysm (proximal point) is noted by a white star, and the center of the curvature just distal to the aneurysm (distal point) is noted by a white square. The aneurysm is noted by two small white arrows, and the neck of the aneurysm is indicated by small white dots. The proximal main inflow (left anterior cerebral artery first segment flow) is indicated by a black arrow, and the distal main outflow (flow from anterior communicating artery to second segment of right anterior cerebral artery) is indicated by a black dotted arrow. The parent artery angle was 121.5° on the first (A) and 101.5° on the last magnetic resonance angiography image (B).

The logistic plot and receiver operating characteristic curve are shown in Figure 2. The parent artery angle change demonstrated a significant relationship with intracranial aneurysm growth, with an area under the curve value of 0.934 and a cutoff value of -3.4°. Representative images of sidewall- and bifurcation-type growing aneurysms are shown in Figures 3-4.

## Discussion

In this study, we identified the narrowing of the parent artery angle as a risk factor for intracranial aneurysm growth. Several other studies have also investigated the association between intracranial aneurysms and surrounding vessel angles. Regarding intracranial aneurysm occurrence and the configuration of the aneurysm vessel, Meng et al. reported that wider angles of the bilateral posterior cerebral arteries led to the redistribution of blood flow to the basilar apex, resulting in lower wall share stress and subsequent development of aneurysms [6]. Baharoglu et al. found that middle cerebral artery (MCA) aneurysms occurred in patients with wider daughter vessel angles, while Tütüncü et al. showed that basilar bifurcation aneurysms occurred in patients with wider bilateral posterior cerebral artery angles [7,8]. Regarding the relationship between intracranial aneurysm rupture and the configuration of the aneurysm vessel, ruptured aneurysms have a wider inflow angle (the angle between the proximal parent artery and the main axis of the aneurysm dome) [4,5,9]. In one of these reports, Lin et al. showed that ruptured aneurysms in the anterior communicating artery had narrow angles between A1 and A2 [5]. In two additional studies based on computational flow dynamics (CFD) analysis, it was reported that the side wall of ruptured aneurysms had a large inflow angle and showed fast blood flow into the aneurysm and that ruptured aneurysms in the posterior communicating artery showed an increase of low wall shear stress area compared to unruptured aneurysms [4,9]. These reports indicate that the configurations of the vessels are closely related to aneurysm occurrence and rupture. Meng et al. [6], Baharoglu et al. [7], Tütüncü et al. [8], Baharoglu et al. [4], Lin et al. [5], Lv et al. [9], and the present study differ in the evaluated vessel and aneurysm angle and in the type of aneurysm development examined. Meng et al. [6], Baharoglu et al. [7], and Tütüncü et al. [8] evaluated two daughter vessel angles of bifurcation-type aneurysms. These reports showed that a wide angle of the two daughter vessels increases the burden of bifurcation and leads to aneurysm occurrence. Baharoglu et al. [4], Lin et al. [5], and Lv et al. [9] evaluated the angle between the proximal artery and the aneurysm axis. These reports showed that a wide angle between the proximal artery and the aneurysm axis increases the vessel flow burden to the aneurysm wall and leads to aneurysm rupture. Our study evaluated the angle of main inflow (aneurysm proximal) and main outflow (aneurysm distal) and showed that a narrowing angle between inflow and outflow increases the burden to the aneurysm and leads to aneurysm growth. A similar angle parameter was used by Lin et al., and this angle is related to aneurysm rupture [5]. Whether a narrow or wide angle affects an aneurysm depends on what part of the angle is evaluated and measured and what development mechanism is investigated.

Regarding the relationship between intracranial aneurysm growth and vessel angle, Boucherit et al. [10] investigated the association between bifurcation aneurysms and the angle of the two daughter vessels (bifurcation angle). They reported that de novo aneurysm formation was observed at wider bifurcation angles and that aneurysm growth is associated with changes in the bifurcation angle, specifically with widening [10]. Miyata et al. [11] showed by CFD analysis that growing MCA aneurysms had a larger aneurysmal inflow rate coefficient (the ratio of the aneurysmal inflow rate to the M1 flow rate) compared to stable aneurysms [11]. In this report, growing MCA aneurysms showed a wider inclination angle between the M1 and M2 arteries [11]. These two articles reported on bifurcation-type aneurysms. In our previous study, narrowing of the parent artery angle was associated with the recurrence of coiled sidewall and bifurcation aneurysms [12]. In addition, the angle of the parent artery, based on the main inflow and outflow arteries, was a useful indicator for both sidewall and bifurcation aneurysms. Similarly, in the present study, we found no significant difference in aneurysm growth between sidewall and bifurcation aneurysms. Aneurysm growth with the narrowing of the parent artery angle was observed in both aneurysm types. Furthermore, growing bifurcation aneurysms with narrowing of the parent artery angle showed widening of the daughter vessel angle.

**Figure 5:**
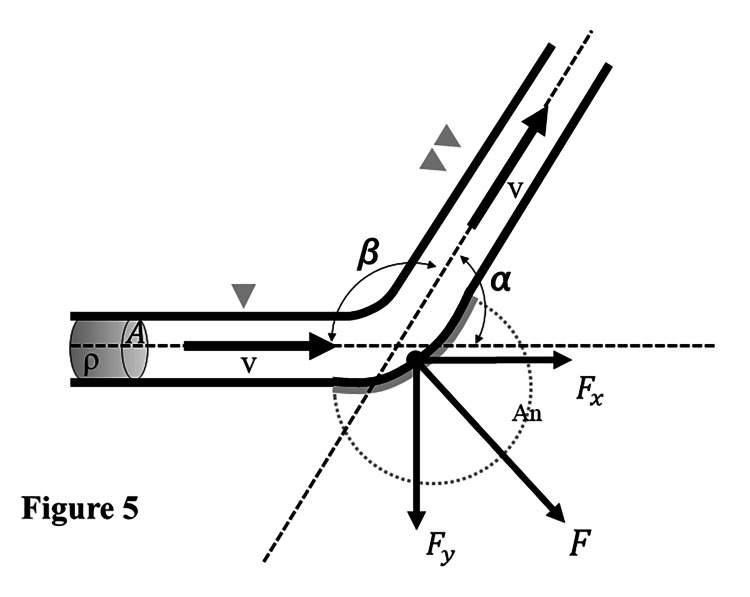
A fluid model for considering the effects of parent artery angle (β) narrowing and the flow impingement zone burden on aneurysms In this model, the aneurysm (An) is indicated by a grey-dotted two-thirds circle. The fluid flow was from the proximal (grey arrowhead) to the distal lumen (double grey arrowheads) through a β-degree curve (angle α is defined as the distal flow angle from X direction for easier physical calculation; angle β is the parameter of this study; α+β=180°). Fluid density was labeled as ρ (g/cm^3^), the cross-sectional area as A (cm^2^), and flow velocity as v (cm/s). The X-axis is parallel to the inflow and proximal lumen, the Y-axis is vertical to the X-axis, and Z is considered as unit width. The flow rate through the cross-sectional area was calculated as Av=Q (cm^3^/s); the mass of fluid per unit time was calculated as ρQ (g); and the momentum of fluid per unit time was calculated as ρQv (g*cm/s). In this model, the change of flow in the X direction is from v to vcosα, and the change of flow in the Y direction is from 0 to vsinα. Hence, the momentum in the X direction is ρQv=ρQvcosα+Fx, and the momentum in the Y direction is ρQvsinα=-Fy. Therefore, Fx=ρQν(1-cosα)  and Fy=-ρQvsinα. Accordingly, F=√(Fx^2^+Fy^2^)=ρQv√((1-cos⁡α )^2^+sin⁡α^2^)=2ρQvsin⁡(α/2), with α=180-β and F=2ρQvsin⁡(α/2)=2ρQvcos⁡(β/2). Based on this formula, narrowing of the parent artery angle (β  becomes smaller) leads to an increase in the burden of the flow impingement zone in the aneurysm and aneurysm neck.

To analyze the influence of the narrowing of the parent artery angle on aneurysms, we employed the fluid model, with the fluid flow direction from the proximal to the distal lumen through a β-degree curve (Figure 5). Based on our calculations, the narrowing of the parent artery angle (β becomes smaller) leads to an increase in the flow power at the aneurysm flow impingement zone and aneurysm neck.

Regarding the effect of widening the parent artery angle on aneurysms, two prior studies have provided useful information. Gao et al. reported that stent-assisted aneurysm embolization, which straightens the parent artery, reduces the flow impingement zone at the aneurysm neck [14]. Ishii et al. reported that straightening of the parent artery by stent-assisted coil embolization above 20° reduces the aneurysm recurrence rate [15]. Considering these findings together with ours, narrowing of the parent artery angle likely increases the inflow burden on the aneurysm neck, leading to aneurysm growth. Understanding blood flow, vessel morphology, and morphological change before treatment may help reduce the incidence of iatrogenic complications or retreatments [4,5,7-12,14-17]. For example, regarding coil embolization of cerebral aneurysms, a patient with a growing aneurysm whose parent artery angle shows narrowing at follow-up may benefit from stent-assisted coil embolization to widen the parent artery angle and, thus, reduce the likelihood of aneurysm recurrence than compared to simple or balloon-assisted coil embolization as these techniques do not affect the parent artery angle [12,14,15]. However, further studies are needed to confirm this.

Regarding the cause behind the narrowing of the parent artery angle, we previously reported that coiled aneurysms migrated in the opposite direction relative to the inflow, reducing the parent artery angle and resulting in aneurysm growth and recurrence [12]. Blood inflow requires time to induce aneurysm migration, allowing for changes to occur in the aneurysm vessel structure that leads to the narrowing of the parent artery angle. Aneurysm migration in a direction opposite to the inflow leads to further narrowing of the parent artery angle, increasing the inflow burden on aneurysms. This may be a vicious cycle of intracranial aneurysm growth in growing unruptured aneurysms. As another possible explanation for the relationship between parent artery angle change and intracranial aneurysm growth, the angle change may initially occur due to arteriosclerosis, leading to aneurysm growth in the further course of the disease. Regardless of the underlying mechanism, there is likely mutual influence between the narrowing of the parent artery angle and intracranial aneurysm growth.

Notably, previously reported risk factors for intracranial aneurysm growth, including age, female sex, previous SAH, hypertension, smoking, and aneurysm size, were not associated with aneurysm growth in the present study [1-3]. However, the findings regarding previous SAH, aneurysm size, and hypertension as aneurysm growth risk factors were contradictory in these studies, and the age at aneurysm growth differed across studies [1-3]. Thus, the role of these risk factors in aneurysm growth remains controversial and requires further studies. In this study, regarding the development of aneurysms, 40 of the 41 growing aneurysms showed main aneurysm body growth with or without bleb development. One growing aneurysm showed a bleb development without the main aneurysm body growth. In this case, the parent artery angle change was -0.2°, and this case showed no recurrence or growth after simple coil embolization. We believe that factors apart from parent artery angle change were involved in this case. Further examination is necessary to understand this.

Several limitations of this study should be acknowledged. First, we used MRA images for analysis. For greater accuracy, the analysis should be based on three-dimensional (3D) computed tomography angiography or 3D rotational angiography images. However, these modalities require contrast media, and 3D rotational angiography adds additional angiography-related risks. Considering the patient’s burden, we used MRA for aneurysm monitoring. Second, we investigated 3D morphological changes as two-dimensional (2D) changes for simplification. As 3D morphological evaluation is more accurate, methods of evaluating the effect of morphological changes of an aneurysm’s parent artery by 3D imaging are under construction. Most current studies have evaluated the surrounding vessel angle’s effect on aneurysms by 2D analyses [4,5,7-11,15]. We think that it is important as the first step to know that the narrowing of the angle of the parent artery observed on 2D imaging affects aneurysmal growth. The parent artery angle and aneurysm were measured from the direction where the proximal (inflow) artery, distal (outflow) artery, and aneurysm were observed in the same plane, as accurately and consistently as possible. This allowed us to detect significant changes in the parent artery angle despite the 2D observation. Furthermore, as 2D images are most commonly used for image analysis, the parent artery angle may be a good and simple parameter for predicting aneurysm growth. Based on this result, we will investigate 3D morphological changes of the parent artery and its effect on aneurysms in a subsequent study. Third, the study design was retrospective, and the sample size was small. This study was performed using untreated aneurysms and simple coiled aneurysms because we thought that narrowing of the parent artery angle would affect these aneurysms by similar mechanisms and that statistical analysis should be performed on these aneurysms as a single group. There were no significant differences in the proportion of simple coiled aneurysms between Groups G and nG. According to univariate analysis, narrowing of the parent artery angle was related to growth in the untreated aneurysms group (30/136, P<0.0001) and in the simple coiled aneurysms group (11/44, P<0.0001). Hence, further prospective studies with larger sample sizes are needed to verify our findings.

## Conclusions

We investigated the relationship between intracranial aneurysm growth and parent artery angle by long-term MRA follow-up data. The results showed that narrowing of the parent artery angle was evident in growing intracranial aneurysms, and this change was identified as a risk factor for intracranial aneurysm growth. Narrowing of the parent artery angle may lead to an increased blood flow burden to the intracranial aneurysm, potentially resulting in a vicious cycle that promotes aneurysm growth. This parameter may be useful for the monitoring of patients with unruptured intracranial aneurysms and may contribute to discerning the mechanism of intracranial aneurysm growth.

## References

[1] Backes D, Rinkel GJ, Greving JP (2017). ELAPSS score for prediction of risk of growth of unruptured intracranial aneurysms. Neurology.

[2] Brinjikji W, Zhu YQ, Lanzino G, Cloft HJ, Murad MH, Wang Z, Kallmes DF (2016). Risk factors for growth of intracranial aneurysms: a systematic review and meta-analysis. AJNR Am J Neuroradiol.

[3] Spencer RJ, St George EJ (2023). Unruptured untreated intracranial aneurysms: a retrospective analysis of outcomes of 445 aneurysms managed conservatively. Br J Neurosurg.

[4] Baharoglu MI, Schirmer CM, Hoit DA, Gao BL, Malek AM (2010). Aneurysm inflow-angle as a discriminant for rupture in sidewall cerebral aneurysms: morphometric and computational fluid dynamic analysis. Stroke.

[5] Lin N, Ho A, Charoenvimolphan N, Frerichs KU, Day AL, Du R (2013). Analysis of morphological parameters to differentiate rupture status in anterior communicating artery aneurysms. PLoS One.

[6] Meng H, Tutino VM, Xiang J, Siddiqui A (2014). High WSS or low WSS? Complex interactions of hemodynamics with intracranial aneurysm initiation, growth, and rupture: toward a unifying hypothesis. AJNR Am J Neuroradiol.

[7] Baharoglu MI, Lauric A, Safain MG, Hippelheuser J, Wu C, Malek AM (2014). Widening and high inclination of the middle cerebral artery bifurcation are associated with presence of aneurysms. Stroke.

[8] Tütüncü F, Schimansky S, Baharoglu MI (2014). Widening of the basilar bifurcation angle: association with presence of intracranial aneurysm, age, and female sex. J Neurosurg.

[9] Lv N, Wang C, Karmonik C (2016). Morphological and hemodynamic discriminators for rupture status in posterior communicating artery aneurysms. PLoS One.

[10] Boucherit J, Kerleroux B, Boulouis G (2023). Bifurcation geometry remodelling of vessels in de novo and growing intracranial aneurysms: a multicenter study. J Neurointerv Surg.

[11] Miyata T, Kataoka H, Shimizu K (2023). Predicting the growth of middle cerebral artery bifurcation aneurysms using differences in the bifurcation angle and inflow coefficient. J Neurosurg.

[12] Yamaguchi S, Ito O, Osaki M (2021). Narrowing of the angle of the parent artery after coil embolization increases the risk for aneurysm recurrence. Clin Neurol Neurosurg.

[13] Hackenberg KA, Algra A, Al-Shahi Salman R (2019). Definition and prioritization of data elements for cohort studies and clinical trials on patients with unruptured intracranial aneurysms: proposal of a multidisciplinary research group. Neurocrit Care.

[14] Gao B, Baharoglu MI, Malek AM (2013). Angular remodeling in single stent-assisted coiling displaces and attenuates the flow impingement zone at the neck of intracranial bifurcation aneurysms. Neurosurgery.

[15] Ishii A, Chihara H, Kikuchi T, Arai D, Ikeda H, Miyamoto S (2017). Contribution of the straightening effect of the parent artery to decreased recanalization in stent-assisted coiling of large aneurysms. J Neurosurg.

[16] Al-Talalwah W (2015). The medial circumflex femoral artery origin variability and its radiological and surgical intervention significance. Springerplus.

[17] Al Talalwah W (2016). A new concept and classification of corona mortis and its clinical significance. Chin J Traumatol.

